# Life-Saving Super-Urgent Liver Transplantation with Replacement of Retrohepatic Vena Cava by Dacron Graft

**DOI:** 10.1155/2010/828326

**Published:** 2010-07-27

**Authors:** Paolo Aseni, Andrea Lauterio, Abdallah Omar Slim, Alessandro Giacomoni, Luca Lamperti, Luciano De Carlis

**Affiliations:** ^1^Department of Surgery, Liver Transplantation Unit, Niguarda “Cà Granda” Hospital, Piazza Ospedale Maggiore 3, 20162 Milan, Italy; ^2^Department of Genaral Surgery, Hepatobiliary and Transplant Unit, Niguarda “Cà Granda” Hospital, Piazza Ospedale Maggiore 3, 20162 Milan, Italy

## Abstract

We describe a modified technique of side-to-side cavo-cavostomy by Dacron interposition prosthesis during a super urgent liver transplantation. A liver graft from a deceased donor was immediately requested on a top priority basis as a consequence of massive bleeding during extended left hepatectomy for a huge hepatic haemangioma arising from the caudate lobe. Veno-venous bypass was employed during anhepatic phase but it was disconnected due to severe fibrinolysis and hypothermia. A porto-caval shunt was performed and the inferior vena cava outflow was restored by a Dacron interposition prosthesis. A liver graft from a deceased donor was available 16 hours later. Due to the shortness of the vena cava of the donor liver graft, the removal of the Dacron graft was impossible and a modified side-to-side cavo-cavostomy between the Dacron interposition graft and the vena cava of the donor liver was than performed. Liver transplantation was uneventful and the patient is doing well 25 months after the surgical procedure. Although the use of synthetic vascular prosthesis should usually be discouraged during organ transplantation, its exceptional use during liver transplantation is possible with long-term good results.

## 1. Introduction

 Uncontrolled bleeding and extensive damage of the liver should be considered a rare but possible complication during elective liver surgery for giant retrohepatic tumours [1–4]. Total vascular exclusion after first-step total hepatectomy followed by *ex vivo *liver surgery is sometimes indicated for huge vascular tumours of the liver characterized by caval and cavo-hepatic junction infiltration. We present here a patient with giant caudate lobe neoplasm who remained anhepatic as a consequence of uncontrollable bleeding during hepatic mobilization. Superurgent liver transplantation was the only way to save the patient's life. 

## 2. Case Report

**Figure 1:**
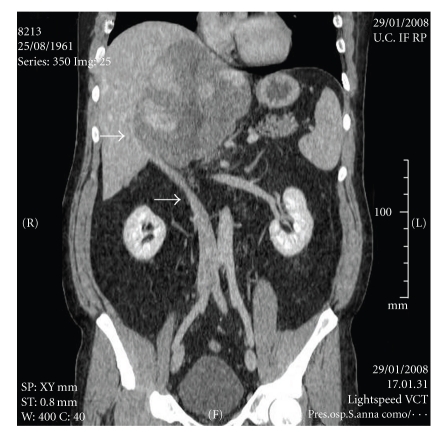
Pre-operative CT scan (coronal view) of the huge hepatic tumour with compression and right dislocation of the interior vena cava (arrows).

**Figure 2:**
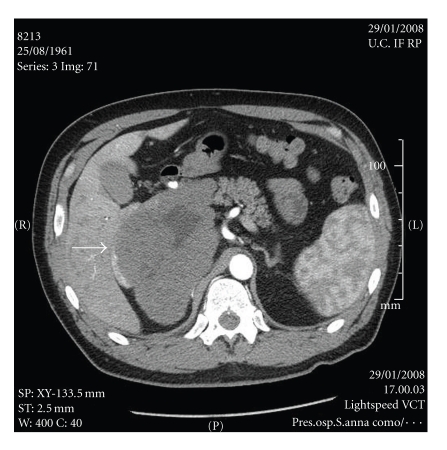
Pre-operative CT scan showing the huge hepatic mass arising from the caudate lobe with a right sided dislocation of the inferior vena cava (arrow).

A 46-year-old male patient with compression and dislocation of the inferior vena cava (IVC) by a giant hepatic haemangioma was referred to our surgical department after two episodes of pulmonary thromboembolism. The second episode occurred while the patient was on oral anticoagulant therapy. Based on abdominal and thoracic CT scan, the patient was diagnosed to have a giant cavernous hepatic haemangioma, arising from the caudate lobe. The tumour was surrounding the inferior vena cava (IVC) and occupying segment I, III, IV, V, and in part segment VIII. A CT scan showed compression of the retrohepatic IVC (Figure 1) with thrombosis of the left and middle suprahepatic veins. On the basis of CT-scan imaging, hepatic malignancy such as haemangiosarcoma could not be ruled out (Figure 2) [5]. 

In order to prevent the risk of further episode of pulmonary embolism, a liver resection was considered. At laparotomy the exploration of the abdomen confirmed the presence of a huge smooth mass, violaceous in colour, occupying the left and the central segments of the liver surrounding and compressing the IVC. An extended left hepatectomy was planned. During the mobilization of the liver, a laceration of the IVC at the junction of right and the middle suprahepatic veins caused an important bleeding which was difficult to control. After Pringle's manoeuvre a running suture by a 3-0 prolene was carried out. The bleeding was controlled only in part. Soon thereafter a worsening of the venous hepatic outflow was evident as suggested by darkening of the whole liver surface. The mass became diffusely hemorrhagic and despite of hilar clamping the bleeding became soon uncontrollable. We decided to proceed to total hepatectomy and to evaluate the feasibility of *ex vivo *bench liver surgery. The suprahepatic and infra-hepatic IVC were cross-clamped. The hilar structures were quickly double ligated and resected. A heparinless veno-venous bypass (VVBP) was inserted from the right femoral vein and from the portal vein to the left axillary vein. The liver was removed and placed in a container with cold Ringer solution. The liver appeared widely damaged by the mass with a retrohepatic caval obstruction. A complete thrombosis of the left and middle hepatic veins was also observed. The huge mass also caused compression of the right hepatic vein that impaired liver flushing trough the portal perfusion. The extensive parenchymal congestion made mandatory to give up each attempt for a left extended hepatectomy by *ex vivo *bench surgery. A request for liver transplantation on a top priority basis was launched in the national and European organ sharing network. 

Through the waiting time for a liver from a deceased donor, the haemostatic condition of the patient worsened as evidenced by a diffuse bleeding all over the operative field. Thromboelastogram showed marked coagulopathy and severe platelet dysfunction. Body core temperature reached the lowest value of 33.6°C. The VVBP was considered at high risk of worsening coagulopathy and hypothermia and it was disconnected. A Dacron interposition prosthesis was inserted to replace the retrohepatic vena cava in order to restore the caval outflow. A porto-caval shunt was also performed by anastomosing end-to-side the recipient portal vein with the Dacron prosthesis. The frozen section histology of the liver excluded hepatic malignancy suggesting liver haemangioma. The relatives of the patient were informed about his dramatic clinical conditions and an informed consent was obtained to proceed to a super urgent liver transplantation. A deceased donor AB0-compatible of a smaller size was available some hours later. The liver graft was harvested and available in our transplant center 12 hours after hepatectomy. 

**Figure 3:**
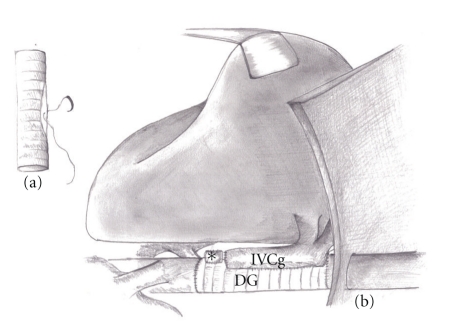
(a) Dacron prosthesis with a wide elliptical incision. (b) Lateral view of the transplanted liver with a wide side-to-side anastomosis between Dacron interposition graft (DG) and the inferior vena cava of the donor liver graft (IVCg) (*portal vein stump).

The porto-caval shunt was disconnected by endovascular stapler. Removal of the Dacron graft was considered but the donor liver was harvested with a caval segment shorter than the Dacron graft. The iliac venous graft harvested from the donor was unsuitable as an extension caval graft. The Dacron prosthesis was then maintained *in situ *and occluded by two vascular clamps. The suprahepatic and infrahepatic vena cava orifices of the donor liver allograft were sutured by endovascular stapler. A wide side-to-side cavo-caval anastomosis was performed between retrohepatic vena cava of the liver and the Dacron interposition graft (Figure 3). The portal vein anastomosis was completed. Clamps removal resulted in a prompt and homogeneous revascularization of the graft. Total anhepatic phase endured 16 hours. Continuity of the arterial and biliary system was re-established. 

**Figure 4:**
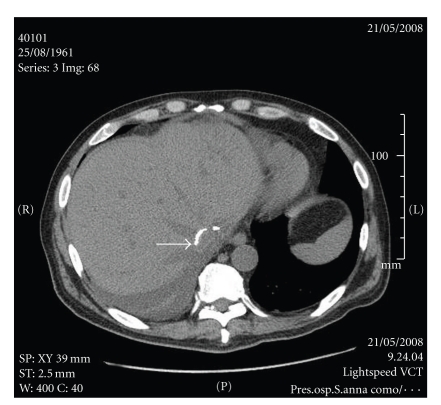
Postoperative CT scan (four months p.o), showing the liver graft with the patent Dacron prosthesis (arrow).

Haemodynamic stability was achieved and maintained throughout the transplant procedure. There were no postoperative complications and the patient was discharged 26 days after the transplant procedure. CT scan 4 months after the transplant procedure showed a normal liver graft with patent Dacron prosthesis (Figure 4). At the moment, after 25 months, the patient is in good physical condition with normal liver function tests. Doppler ultrasound studies at 3 and 15 months showed perfect permeability of cavo-cavostomy and patency of the Dacron graft. 

## 3. Discussion

Emergency liver transplantation as a consequence of a massive bleeding during elective liver surgery for tumours infiltrating the IVC, is a rare dramatic clinical condition. Surgery of the caudate lobe, due to its unique anatomical site, is considered at high risk of intraoperative complications even by experienced surgeons [6] with a perioperative mortality ranging from 2% to 9% rate [1–4]. Exvivo bench surgery can be a surgical option in difficult cases [7]. However, we were unable to flush the liver at bench surgery and superurgent liver transplantation was the only available option. Although with a size mismatch, an AB0 compatible liver graft was soon available. During the anhepatic phase, a severe coagulopathy and hypothermia contraindicated the prolonged use of the VVBP by centrifugal pump. The restoration of the caval integrity was carried out by a Dacron interposition prosthesis to prevent renal failure, splanchnic congestion and to restore cardiac function [8, 9]. The vena cava of donor liver was shorter than the Dacron interposition graft as a result of the size mismatch between the donor and the recipient. Dacron prosthesis has been sometimes employed for caval reconstruction during resection of huge liver tumours [2]. 

A synthetic prosthesis as a bridge for a short vena cava during liver transplantation has recently been reported; however, the same authors reported a severe anastomotic stenosis occurring six months after operation [10]. Our technique is a modification of the side-to-side cavo-cavostomy previously described by Belghiti et al. [11] and Lerut and Gertsch [12]. It simply differs from the original technique for the utilization of the Dacron prosthesis. 

In conclusion, superurgent liver transplantation should be considered a rescue procedure in selected patients when challenging liver resections are carried out. 

Synthetic vascular graft should not be recommended during organ transplantation for the high risk of infection and graft thrombosis. However, we were able to demonstrate that, in difficult situations when technical and anatomical difficulties threaten the completion of the liver transplant procedure, the utilization of Dacron prosthesis can be considered a viable option with long term good results.

## References

[1] Wen Z-Q, Yan Y-Q, Yang J-M, Wu M-C (2008). Precautions in caudate lobe resection: report of 11 cases. *World Journal of Gastroenterology*.

[2] Hemming AW, Reed AI, Langham MR, Fujita S, Howard RJ (2004). Combined resection of the liver and inferior vena cava for hepatic malignancy. *Annals of Surgery*.

[3] Fan J, Wu Z-Q, Tang Z-Y (2001). Complete resection of the caudate lobe of the liver with tumor: technique and experience. *Hepato-Gastroenterology*.

[4] Hu J-X, Miao X-Y, Zhong D-W, Dai W-D, Liu W (2005). Anterior approach for complete isolated caudate lobectomy. *Hepato-Gastroenterology*.

[5] Itai Y, Teraoka T (1989). Angiosarcoma of the liver mimicking cavernous hemangioma on dynamic CT. *Journal of Computer Assisted Tomography*.

[6] Chaib E, Ribeiro MAF, Silva FDSC, Saad WA, Cecconello I (2008). Caudate lobectomy: tumor location, topographic classification, and technique using right- and left-sided approaches to the liver. *American Journal of Surgery*.

[7] Hemming AW, Cattral MS (1999). Ex vivo liver resection with replacement of the inferior vena cava and hepatic vein replacement by transposition of the portal vein. *Journal of the American College of Surgeons*.

[8] Baumgartner F, Scudamore C, Nair C, Karusseit O, Hemming A (1995). Venovenous bypass for major hepatic and caval trauma. *Journal of Trauma*.

[9] Fan ST, Yong BH, Lo CM, Liu CL, Wong J (2003). Right lobe living donor liver transplantation with or without venovenous bypass. *British Journal of Surgery*.

[10] Jeon H, McHugh PP, Banerjee A, Gedaly R, Johnston TD, Ranjan D (2008). The use of Dacron graft for cavo-cavostomy in orthotopic liver transplantation. *Transplantation*.

[11] Belghiti J, Panis Y, Sauvanet A, Gayet B, Fékété F (1992). A new technique of side to side caval anastomosis during orthotopic hepatic transplantation without inferior vena caval occlusion. *Surgery Gynecology and Obstetrics*.

[12] Lerut J, Gertsch P (1993). Side-to-side cavo-cavostomy: a useful aid in “complicated” piggy-back liver transplantation. *Transplant International*.

